# Diagnostic Accuracy and Applicability of a PCR System for the Detection of *Schistosoma mansoni* DNA in Human Urine Samples from an Endemic Area

**DOI:** 10.1371/journal.pone.0038947

**Published:** 2012-06-11

**Authors:** Martin Johannes Enk, Guilherme Oliveira e Silva, Nilton Barnabé Rodrigues

**Affiliations:** 1 Laboratório de Esquistossomose, Centro de Pesquisas René Rachou, FIOCRUZ, Belo Horizonte, Minas Gerais, Brazil; 2 Laboratório de Parasitologia, Universidade Vale do Rio Doce, Governador Valadares, Minas Gerais, Brazil; 3 Laboratório de Imunologia Celular e Molecular, Centro de Pesquisas René Rachou, FIOCRUZ, Belo Horizonte, Minas Gerais, Brazil; The George Washington University Medical Center, United States of America

## Abstract

Schistosomiasis caused by *Schistosoma mansoni*, one of the most neglected human parasitoses in Latin America and Africa, is routinely confirmed by microscopic visualization of eggs in stool. The main limitation of this diagnostic approach is its lack of sensitivity in detecting individual low worm burdens and consequently data on infection rates in low transmission settings are little reliable. According to the scientific literature, PCR assays are characterized by high sensitivity and specificity in detecting parasite DNA in biological samples. A simple and cost effective extraction method for DNA of *Schistosoma mansoni* from urine samples in combination with a conventional PCR assay was developed and applied in an endemic area. This urine based PCR system was tested for diagnostic accuracy among a population of a small village in an endemic area, comparing it to a reference test composed of three different parasitological techniques. The diagnostic parameters revealed a sensitivity of 100%, a specificity of 91.20%, positive and negative predictive values of 86.25% and 100%, respectively, and a test accuracy of 94.33%. Further statistical analysis showed a *k* index of 0.8806, indicating an excellent agreement between the reference test and the PCR system. Data obtained from the mouse model indicate the infection can be detected one week after cercariae penetration, opening a new perspective for early detection and patient management during this stage of the disease. The data indicate that this innovative PCR system provides a simple to handle and robust diagnostic tool for the detection of *S. mansoni* DNA from urine samples and a promising approach to overcome the diagnostic obstacles in low transmission settings. Furthermore the principals of this molecular technique, based on the examination of human urine samples may be useful for the diagnosis of other neglected tropical diseases that can be detected by trans-renal DNA.

## Introduction

Schistosomiasis is from a global public health perspective one of the most important water-borne parasitosis and a major neglected tropical disease, with more than 200 million people infected and close to 800 million at risk [1]. The disease burden is estimated to exceed 70 million disability-adjusted life-years [2]. Although in the specific case of *Schistosoma mansoni* substantial progress has been made in the control of this disease in Egypt and Latin America by reducing morbidity and prevalence, transmission continues, and the disease has spread to previously non-endemic areas [3]–[5]. Under these circumstances, characterized by low prevalence and infection intensity, the routinely used diagnostic tool, based on the detection of parasite eggs in stool, the Kato-Katz technique [6], lacks sensitivity to identify reliably positive cases [7]–[9]. Consequently the prevalence in these settings is significantly underestimated and undetected carriers maintain and spread the disease, hampering further progress of the control efforts [10]. In order to overcome the intrinsic limitation of this technique the examination of various stool samples is necessary, neutralizing all operational advantages of this method [11]–[13]. As alternative, serological tests can be applied together with or without coproscopic techniques [14]. Available tests present either low sensitivity, cross-reactivity with other helminth infections or cannot distinguish between active and past infections, which is particularly important for endemic areas [15], [16]. New candidates for serological diagnostic of schistosomiasis, partly derived from proteomic analysis, are still in the experimental phase and require further improvement and validation [17]–[20]. Also all these methods require collection of blood, an inconvenience for their application at large scale [15].

Considering the above mentioned and the yearly rising numbers of schistosomiasis infected travelers and migrants, who in general showing very low infection intensity in its early stage, more sensitive methods for the diagnosis of this disease, are urgently needed [16], [21], [22].

Diagnostic techniques based on the Polymerase Chain Reaction (PCR) rely on the detection of *Schistosoma* spp. DNA in feces, serum [23]–[27], plasma [28] and urine [29] and have shown high sensitivity and specificity. In all cases DNA extraction methods are based on the use of organic solvents or commercial kits making the process hazardous and/or expensive to work with a large number of samples. Recently, a cheap salting out and resin method for DNA extraction from urine samples was described [30], and in this study its application in combination with a conventional PCR technique was evaluated. The diagnostic performance of this test in comparison to a reference test, obtained through coproscopical examination of four stool samples, in a Brazilian endemic area for *S. mansoni* is presented.

## Materials and Methods

### Study Population

**Figure 1:**
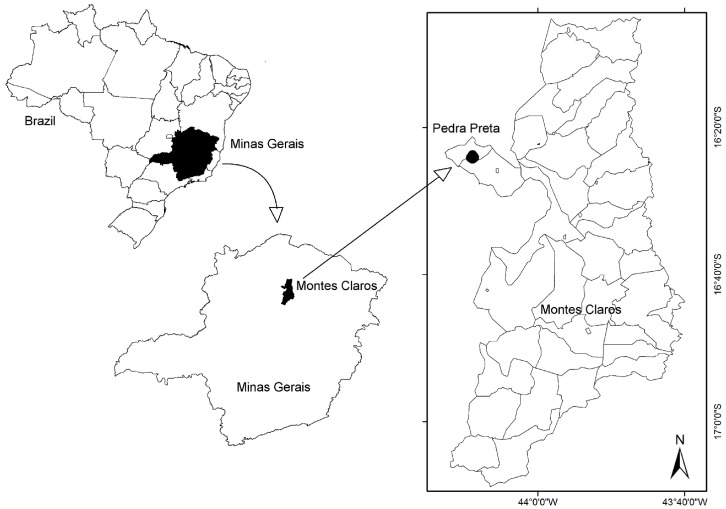
Geographical illustration of the study area situated in the locality of Pedra Preta, municipality of Montes Claros, state of Minas Gerais, Brazil.

**Figure 2:**
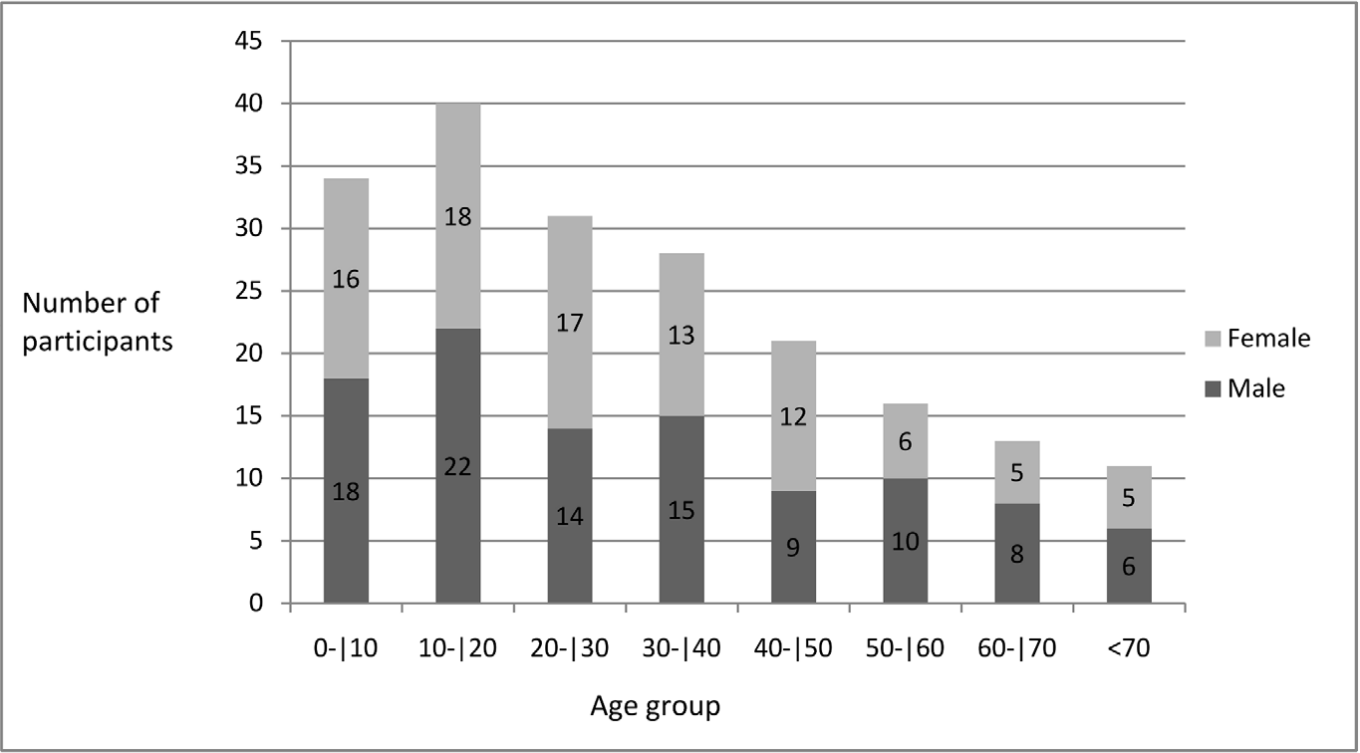
Distribution according to gender and age groups of the study population living in Pedra Preta, Minas Gerais, Brazil.

The study had been conducted in Pedra Preta, a small village in the north of the state Minas Gerais, Brazil, known as endemic area for *S. mansoni* (Figure 1). All 214 villagers were invited to participate in this study and 194 provided the stool and urine samples required for this study. The study population of 194 participants was composed of 92 females and 102 males, of whom 66 were under 18 years of age (32 females and 34 males between 1 and 17 years old). The 128 adults included 60 females and 68 males between 18 and 86 years old. More detailed information about age groups and gender is given in Figure 2. The study protocol and objectives were explained to the participants and written informed consent was obtained from all adults and from a parent or legal guardian of minors, in compliance with the guidelines of the Helsinki Declaration about research carried out on humans. The study received approval by the Ethical Review Board of the René Rachou Research Center / FIOCRUZ - MINAS (No. 03/2008) as well as by the Brazilian National Ethical Review Board (CONEP - No 784/2008). Individuals with positive stool examinations for schistosomiasis were treated with praziquantel and those positive for other helminth infections with albendazole as recommended by the Brazilian Ministry of Health.

### Stool Examinations

The participants were asked to provide a total number of four stool samples collected on consecutive days. Twelve Kato Katz (KK) smears were prepared from the first sample and two smears from the second, the third and the forth, respectively, resulting in 18 slides for each participant. The cumulative results of all KK slides per participant combined with two other parasitological methods, namely the Saline Gradient technique, using 500 mg feces from the first sample and the Miracidia Hatch test using 1 g feces from the first sample, served as combined reference test (gold standard) for comparisons. Both techniques are described in detail elsewhere [31], [32]. The KK slides as well as the material obtained from the other techniques were examined by three experienced laboratory technicians.

### Urine Examinations

All participants provided one urine sample of 10 ml of morning urine collected at the same day as the first stool sample. The samples were received in the field laboratory and stored in a common freezer and transported frozen to the Rene Rachou Research Center for further processing. The PCR assays were conducted and read without previous knowledge of the results obtained by microscopic examinations of the stool samples, which were carried out by another team.

DNA extraction from the urine samples was carried out as recently described by Enk et al [30]. The urine samples were defrosted, added EDTA to a final concentration of 40 mM and homogenized by tube inversion. An amount of five mL of each sample was transferred to a 15 mL Falcon tube and heated in a water bath at 100°C for 10 min. Five hundred micro liters of 5 M NaCl was added to each tube. The tubes were shaken vigorously for 15 sec and placed on ice for 1 hr.

After centrifugation for 10 min at 1,700×g the supernatant was transferred to another tube. Absolute ethanol, in a quantity of two times the sample volume, was added, and samples were kept at −20°C for 2 hours for DNA precipitation. The tubes were shaken vigorously for 15 sec and centrifuged at 1,700×g for 10 min. The pellet was resuspended in 200 µL 70% ethanol, transferred to a 0,5 mL microcentrifuge tube and centrifuged at 20,000×g for 20 min. The pellets were dried and resuspended in 100 µL of DNAse free water and 100 µL of InstaGene matrix® (BioRad). The samples were incubated at 56°C for 30 min and 100°C for 8 min, vortexed at high speed for 10 sec and centrifuged at 20,000×g for 3 min, the supernatant transferred to a new tube and used as template for PCR.

The PCR primers were previously designed by Pontes et al. [23], to amplify a fragment of 110 bp from a highly repeated 121-basepair sequence of *S. mansoni* reported by Hamburger et al,1991(GenBank accession # M61098) [33] that comprises about 10% of the parasite genome (600,000 copies per cell). As urine samples contain alongside with human DNA also bacterial, yeast and in case of our study expected *S. mansoni* DNA, all samples were diluted to a total DNA concentration of 2 ng/µL. For amplification, 2 µL of the extracted DNA from the urine samples served as template. PCR was carried out with a final volume of 10 µL using 0,8 U of GoTaq DNA polymerase, 1 µL of STR 10× buffer (Promega), 0.1 µg/µL of 1× BSA, 0.5 pmol of each, forward (5′ GATCTGAATCCGACCAACCG 3′) and reverse (5′ ATATTAACGCCCACGCTCTC 3′) primers, and enough water to complete the final volume. A total of 40 amplification cycles were conducted, each of them consisting of three steps: 30 sec denaturation at 95°C, 30 sec annealing at 65°C and 30 sec extension at 72°C. As PCR positive controls, 1 ng of *S. mansoni* adult worm DNA was used as template. PCR negative controls containing all elements of the reaction mixture and water instead of DNA were also included in each PCR assay as surveillance for contamination. PCR assays were conducted 3 times for each sample.

**Figure 3:**
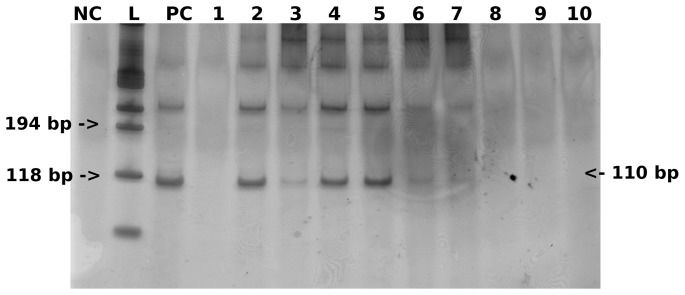
Visualization of 12 PCR assays with silver stained 8% polyacrylamide gel, showing the expected *S. mansoni* 110 bp DNA fragment in positive urine samples. NC: negative control; L: 100 bp Ladder; PC: positive control; lines 2, 3, 4, 5, 6, and 7: *S. mansoni* positive urine samples; lines 1, 8, 9, and 10: *S. mansoni* negative urine samples.

Electrophoresis of 3 µl of the amplified samples was carried out in 8% polyacrylamide gel using a Mini-Protean II (Bio-Rad, Hercules, CA). The gel was then silver stained [34] and the images were recorded by digital photography (Figure 3).

### Statistical Analysis

Only data from participants who provided sufficient biological material to perform the parasitological examinations for the combined reference test and the PCR from the urine samples were used to analyze the agreement between these two diagnostic methods, and to calculate the sensitivity, specificity, positive predictive value (PPV) and negative predictive value (NPV) of PCR in urine samples. The comparative analysis for the performance of the urine PCR in relation to the combined reference test, and the calculation of the sensitivity, specificity, PPV, NPV, and *k* index were done using the OpenEpi Version 2.3 program [35]. The *k* index was interpreted according to Landis and Koch [36] indicating excellent agreement between 1.00 and 0.81, good between 0.80 and 0.61, moderate between 0.60 and 0.41, weak between 0.40 and 0.21 and negligible between 0.20 and 0.00.

## Results

### Stool and Urine Examinations

Stool examinations according to the KK technique revealed 22 (11.34%) schistosomiasis positives for 1 slide, 27 (13.92%) for two and 38 (19.6%) for 12 of the first stool sample. Including the results of two slides from the second, third and fourth stool samples, the number of positives increased from 46 (23.71%) to 58 (29.90%) and 64 (32.99%), respectively. The Saline Gradient technique and the Miracidia Hatch test detected one and four additional positives, which were not identified by the KK method, resulting in a total of 69 (35.57%) infected. This value was considered as reference for further comparisons.

The PCR technique applied in the urine samples identified 80 of the 194 participants as positive for schistosomiasis, indicating an infection rate of 41.24%.

### Comparison between the PCR Technique and the Reference Test

The comparison of the results obtained by the *Schistosoma* PCR assay of urine samples and the combined reference test as described above is shown in Table 1.

**Table 1:** Comparison of positive and negative results obtained by PCR in urine samples and the copro-parasitological reference test for the detection of *S. mansoni* infection.

	Reference test		
PCR urine	positive	negative	total
positive	69	11	80
negative	0	114	114
total	69	125	194

Diagnostic parameters were calculated revealing a sensitivity of 100% for the PCR assay in urine samples, as none of the positives found by the combined reference test remained undetected. The specificity of the molecular technique was high with a value of 91.20% (CI (95%): 84.93–95.02). Positive and negative predictive values showed figures of 86.25% (CI (95%): 77.03–92.15) and 100% (CI (95%): 96.74–100), respectively. The test accuracy was 94.33% (CI (95%): 90.13–96.8). Further statistical analysis showed a *k* index of 0.8806, indicating an excellent agreement between the two tests.

## Discussion

The results of this study clearly show the significant underestimated schistosomiasis infection rate in this setting and confirm data published in another study of our group carried out under similar conditions [10]. The 3.14 fold underestimation of the number of positives, based on the parasitological techniques and 3.64 fold of the molecular method, may explain why control efforts in areas with prevalence lower than 15% are less successful. The fact that most of the control strategies are based on the examination of one or two KK slides of a single stool sample to identify and treat infected, implicates, according to this data, that only one out of three schistosomiasis positive individuals is treated. At least 47 positives or 24.25% of the total population of the village continue to maintain the disease in this small area and remain at risk to develop chronic and ectopic forms, especially schistosomal myeloradiculopathy, as well as indirect pathology and morbidity of the disease, which may occur even among patients with low individual worm burden [15], [37], [38]. This evidence emphasizes once more the urgent need of an accurate diagnostic tool for individual patient management and for possible guidance of community based control efforts in low transmission areas.

Since the discovery of cell-free nucleic acids circulating in blood and being partially excreted into urine, this field has advanced and has produced many exciting new developments [39]. Many publications demonstrate that these nucleic acids could be used for development of clinical diagnostic PCR-based assays in the areas of prenatal genetic testing, oncology, organ transplantation, and, important in this context, infectious and parasitic diseases [40]. DNA fragments from cells that have died throughout the body appear in the urine, presenting relatively low molecular size fragments (100–200 bp) which should be considered when deciding on methods of DNA extraction and PCR design for analysis. In particular, if PCR is used for amplification and detection of DNA sequences, as in our case of *S. mansoni*, the application of a small sized amplicon significantly enhances sensitivity. Therefore a pair of primers targeting a sequence of 121 bp was used for PCR, which has been applied extensively in *S. mansoni* diagnostics [23], [26], [27]. With regards to the extraction of transrenal schistosomiasis DNA, a technique recently described by our group, which is based on a salting-out and resin procedure, was applied for the first time among a significant number of human samples from an endemic area [30]. It reaches an analytical sensitivity of 1.28 pg of parasite DNA per mL urine. Since the genome of *S. mansoni* contains about 580 fg DNA per cell, our PCR system can, theoretically, detect DNA corresponding to two cells of the multicellular parasite, per mL urine [41]. This analytical sensitivity proofed to be sufficient for following molecular analysis of urine samples of individuals from an endemic area.

As table 1 shows, the urine PCR technique did not miss a single case of schistosomiasis, detected by the reference test, which justifies the sensitivity of 100%. The discordant results of 11 positives identified by PCR and missed by the reference test, explains the high specificity of 91.20% (95%CI: 84.93–95.02). It is worth to note here, that the reference test is of paramount importance for the evaluation of a new diagnostic test. There exists a general consensus that parasitological tests have an assumed specificity of 100% but cannot ensure the same values for sensitivity [42], [43]. In order to establish a more robust sensitivity of the reference test, three different methods were chosen in this study, which allowed an exhaustive analysis of the fecal samples. Even so it is evident that not all schistosomiasis positives were detected, due to the well recognized daily variation of individual parasite egg output and uneven distribution of the eggs in the stool [44], [45]. Considering the well accepted compromise between accuracy and cost, consisting of three different stool samples, each examined by two KK slides, 15 positives would have been missed by this approach, and the number of discordant urine PCR examinations would have increased from 11 to 26, presenting a decreased artificial specificity of 81.43% (95%CI: 74.18–87.00), PPV of 67.50% (95%CI: 56.64–76.76) and diagnostic accuracy of 86.6% (95%CI: 81.09–90.69) [46], [47]. This example demonstrates two important issues. First, the dilemma of establishing a standard for a reference test which could be applied for the evaluation of new high specific and sensitive diagnostic techniques for schistosomiasis, resulting in uncertainty about the determination of discordant or so called “false positive results” obtained with the new technique. Second, it seems to be likely that cell free DNA as well as trans-renal DNA levels are less subjective to fluctuation and variation as long as the infection remains. A proof for this assumption is the fact that the examination of a single urine sample collected on the same day as the first stool sample detected all positives identified by the reference test, which is based on three different techniques and examining stool samples of four consecutive days.

The lack of sensitivity of the reference test might not be the only explanation for the discordant results. As published by Sandoval et al. [48], *Schistosoma* DNA in the urine of mice had been detected by PCR only one week after infection with cercariae. These results were confirmed by data from an experimental study in 15 Swiss mice using our PCR system, which showed that all mice proofed positive for infection after five days [49]. Given these findings, it is probable that the PCR system detects infection in humans during the larval stage or at least before parasite eggs can be found in the stool, i.e. 30 to 40 days after infection, which can explain the discordant results. Another remote but plausible reason for discordant results could be unisexual infection with male worms, which gains more significance in low transmission settings [9]. Finally, contamination of the samples during the handling procedures must be considered. Therefore high standards of quality control, such as physical barriers (separation of rooms and materials, use of bleach and laminar flow chamber with UV light) were applied during the procedure. Furthermore contamination as explanation for the discordant results in this case seems to be very unlikely, as negative controls showed no positive outcome.

Finally this urine based PCR system is characterized by certain operational advantages. The collection of urine samples stands out as the most important aspect. In the study described here, all 194 morning urine samples were collected during the first two consecutive days in the field. In contrast to that, the collection of the four stool samples, necessary for the reference test, took 12 days. This difference in time for sample collection reduces costs, principally in the sector of human resources. Another advantage of this technique is the high diagnostic throughput. One well-trained technician is able to process 96 urine samples per day, including extraction, amplification and visualization, of which extraction is the most work intensive process. Compared to the KK technique a number of 60 to 70 slides per day, including preparation and examination is a reasonable quantity for one well trained technician, not considering the significant loss of diagnostic accuracy of a single KK slide per sample, as lined out above. Focusing on high sensitivity and specificity, particularly important in areas of low transmission of the disease, the diagnostic through-put of the urine PCR system, as of other PCR systems as well, also indicates to decrease indirect costs. Again the reduction affects the sector of human resources, as clearly less time is necessary to process the 96 urine samples than the processing of 1728 KK slides, composed of 18 slides of four different stool samples collected from each of 96 patients, for accurate schistosomiasis diagnostic as shown in this study.

Estimates of the direct costs of the described urine PCR system including reagents and consumables are less than US$4.00 per examination (extraction – US$0.70, amplification – US$2.70 and visualization – US$0.20), and there is still room for cost reduction by optimizing the extraction and amplification procedures of the system. In this context, it is worthwhile to note that our study, besides evaluating the technique in comparison to the routinely-used coproscopic method, also intended to minimize costs. This intent led to the decision to give preference to conventional PCR over the qPCR technique. Besides much higher costs for sample examination, the qPCR technique requires much more sophisticated equipment which is, to our knowledge, not available in mid-level laboratory facilities of schistosomiasis endemic countries and is limited to research institutions and high-level tertiary care centers. One might argue that qPCR, although much more expensive and less available, provides the advantage of not only identifying the presence of trans-renal DNA in samples, but also quantifying it and thereby indirectly estimating parasite burden. In our view this advantage of qPCR loses importance in relation to the approach presented in this paper when it is applied in areas of low transmission with the majority of infected individuals having low worm burdens (e.g., less the 100 epg), and where the routinely used Kato-Katz technique lacks sensitivity. Therefore the focus of our study was on the detection of positives while keeping costs as low as possible. The same considerations, namely the high costs and less compelling need for quantification, account for why the PCR-ELISA system described by Gomes et al was not used in this study [27].

In conclusion the described PCR system using urine samples for the detection of *S. mansoni* provides a promising alternative and additional powerful technique for the diagnosis of the disease. The ease of sample collection and the high sensibility and specificity of this system indicate its usefulness and value for the identification of schistosomiasis infections in low transmission settings and among individuals with low worm burden, which is especially important in the context of transmission control and disease surveillance. Its potential for the diagnosis of infections in the early stage opens a new perspective for patient management that requires further investigation.
